# A Statistical Framework for the Adaptive Management of Epidemiological Interventions

**DOI:** 10.1371/journal.pone.0005807

**Published:** 2009-06-05

**Authors:** Daniel Merl, Leah R. Johnson, Robert B. Gramacy, Marc Mangel

**Affiliations:** 1 Department of Statistical Science, Duke University, Durham, North Carolina, United States of America; 2 Department of Ecology, Evolution, and Marine Biology, University of California Santa Barbara, Santa Barbara, California, United States of America; 3 Statistical Laboratory, University of Cambridge, Cambridge, United Kingdom; 4 Center for Biomolecular Science and Engineering and Department of Applied Mathematics and Statistics, University of California Santa Cruz, Santa Cruz, California, United States of America; Fred Hutchinson Cancer Research Center, United States of America

## Abstract

**Background:**

Epidemiological interventions aim to control the spread of infectious disease through various mechanisms, each carrying a different associated cost.

**Methodology:**

We describe a flexible statistical framework for generating optimal epidemiological interventions that are designed to minimize the total expected cost of an emerging epidemic while simultaneously propagating uncertainty regarding the underlying disease model parameters through to the decision process. The strategies produced through this framework are adaptive: vaccination schedules are iteratively adjusted to reflect the anticipated trajectory of the epidemic given the current population state and updated parameter estimates.

**Conclusions:**

Using simulation studies based on a classic influenza outbreak, we demonstrate the advantages of adaptive interventions over non-adaptive ones, in terms of cost and resource efficiency, and robustness to model misspecification.

## Introduction

Epidemiological interventions generally remove susceptible individuals or apply some form of treatment to infected individuals in order to prevent further spread of a disease. The susceptible population may be culled, as in the case of foot-and-mouth disease [1], [2], in which case the total population size is permanently reduced. The infected population may be quarantined, as in the case of SARS [3], in which case total population size is unchanged but the fraction of infecteds that may be in contact with susceptibles is reduced. Most commonly, susceptibles are vaccinated (cf influenza or smallpox [4], [5]), in which case the total number of susceptibles, but not the total population size, is reduced.

Each of these interventions incurs a quantifiable cost: culling results in additional deaths; medical treatments or quarantines result in monetary expenses; vaccination incurs both monetary expenses, and in some cases additional vaccine-induced infections. Additionally, in many situations the costs associated with each of these actions can depend upon the state of the disease within the population of interest. For example, per-dosage prices of vaccine can increase as resources become scarce as a result of an aggressive vaccination campaign. Similarly, vaccine efficacy can decrease as a result of selection for drug resistance. Such observations raise the question of how to find optimal interventions that adaptively depend on the state of the epidemic.

A key challenge to calculating optimal intervention strategies involves devising ways to characterize and explore the space of intervention policies. Most existing work on optimal intervention has required various limiting assumptions about the forms of such strategies. Ball and Lyne [6] considered optimal vaccination in terms of the allocation of vaccine doses to households of various sizes in an explicitly structured population model. Patel *et al* [7] considered optimal vaccination in terms of the allocation of vaccine doses to different age classes in an explicitly age- and geographically- structured population model. Tildesley *et al* [1] describe optimal vaccination strategies for a foot-and-mouth epidemic in which the optimized parameter is the size of the radius surrounding a point of infection within which all livestock are to be vaccinated. These methods are primarily concerned with pre-emptive interventions that can be completed before the arrival of the pathogen. Under such scenarios, there is no need to consider adaptive or sequentially updated interventions because as soon as the intervention policy is triggered, the threat of epidemic is eradicated. In real scenarios, such widespread vaccination may not be achievable. Moreover, these methods traditionally involve calculations that assume no uncertainty in key model parameters such as transmission rate, recovery rate, and mortality rate. Recently Elderd *et al* [8], using Bayesian methods, demonstrated the importance of explicitly quantifying such underlying uncertainty when forecasting the expected efficacy of trace versus mass vaccination policies. Their findings demonstrate that accurate propagation of parameter uncertainty can sometimes reveal deep and troubling consequences of a proposed vaccination strategy, and they suggest that incorporation of such uncertainty could impact policy decisions.

Here we address the question of how to dynamically propagate uncertainty in order to respond to an emerging epidemic while simultaneously and continuously learning about its underlying transmission dynamics. Estimation of model parameters is facilitated by regarding the transmission dynamics as stochastic processes rather than deterministic solutions to a structural equation model. This allows us to explicitly account for uncertainty in both model parameters and disease transmission. We consider a very general class of vaccination strategies defined by a fraction of the current susceptible population to be targeted for vaccination, and a threshold number of susceptibles such that once the number of susceptibles falls below this threshold, the vaccination campaign is called off. We demonstrate the calculation and application of optimal strategies of this form when coupled with iteratively updated parameter estimates using simulations based on a well-studied influenza outbreak [9]. Our emphasis is not on the realism of the underlying SIR model (though it has been shown that even simplistic transmission models can provide good fit to actual data [10]), but rather to describe an effective approach for combining estimation and policy calculation. Permitting greater flexibility in the form of the possible intervention renders calculation of optimal intervention strategies analytically intractable, thus requiring evaluation by Monte Carlo-based methods. Once in a Monte Carlo-based framework, it becomes straightforward to couple the evaluation of intervention strategies with Bayesian procedures for performing on-line estimation of parameters of the underlying epidemic model, thereby propagating parameter uncertainty through to policy decisions.

The policies produced by this framework are optimal in that they minimize the expected cost of the epidemic and adaptive in that the optimal policy changes as a function of the state of the epidemic and the degree of uncertainty in underlying model parameters. Using extensive simulation studies we compare the distribution of costs accrued under adaptive intervention to those arising from non-adaptive policies in a variety of scenarios. Our studies show that adaptive policies perform similarly to nonadaptive policies based on perfect parameter estimates, and significantly better than nonadaptive policies based on imperfect parameter estimates. Additionally, we show that adaptive online estimation affords the method some robustness to model misspecification. These results further demonstrate the importance of accounting for such underlying uncertainties in dynamic settings and indicate the utility of adaptive policies in settings where perfect estimates and a true model do not exist. All computational methods used herein have been made freely available through the amei (Adaptive Management of Epidemiological Interventions) R package [11].

## Results

A classic study of Murray's [9] describes the spread of influenza through the population of a British boarding school. During the course of the epidemic, which was traced to the arrival of a single infectious student, all 763 students were eventually infected. The epidemic conforms to many standard assumptions of SIR models: a population essentially closed to immigration and emigration, recovery with immunity, and nearly homogeneous mixing of susceptibles and infectives.

Viewing the transmission dynamics as a discrete time stochastic process rather than a deterministic system of coupled differential equations implies a distribution of possible outcomes for the epidemic. By conditioning on parameter values and initial conditions (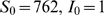), Monte Carlo simulation can be used to explore the distributions of numbers of susceptible, infected, and recovered individuals, as well as total accrued cost, as functions of time. Murray provides estimates of the transmission rate (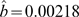) and recovery rate (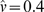), which we regard as the “true” underlying parameter values in our simulations. Additional aspects of the transmission function are discussed in the Methods section. We assume that all costs can be expressed in a common monetary cost unit. Other choices of cost functions that address the issue of nonconformable costs (e.g. lives vs dollars) are mentioned in the Discussion.

**Figure 1:**
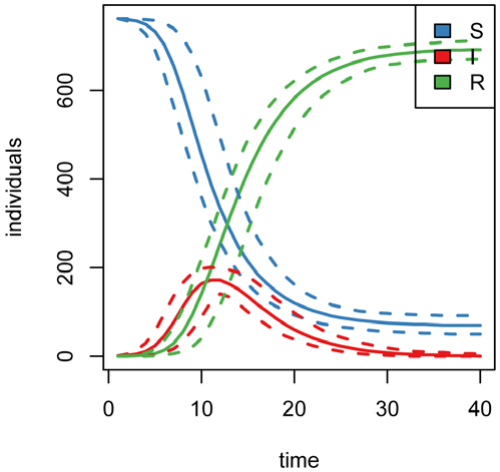
Simulated epidemics. (2.5,50,97.5)-% quantiles for numbers of susceptible, infected, and recovered individuals over 1000 simulations of the epidemic without intervention.

**Figure 2:**
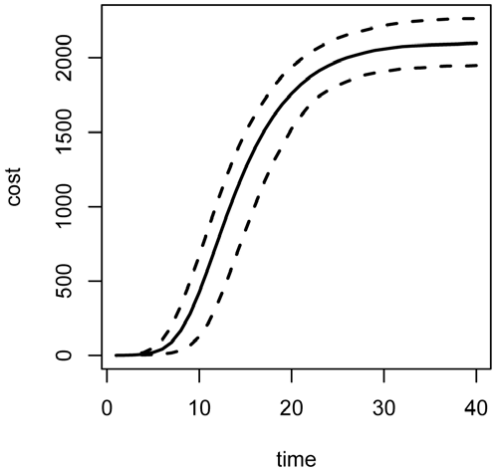
Expected costs under nonintervention. (2.5, 50, 97.5)-% quantiles for total cost accrued over 1000 simulations of the epidemic without intervention. The mean total cost after 40 days is 2100 cost units, with quantile bounds (1949,2263).

Setting the unit cost to be that of maintaining a single infected individual for one time step (cost per infected, 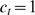), repeated forward simulation of the epidemic (Figure 1) indicates that the mean total cost over 40 time steps is approximately 2100 cost units (Figure 2), attributable entirely to the cumulative cost of maintaining a large population of infected individuals until recovery.

### Variable Stop Time Vaccination

We consider a relatively simple but flexible class of intervention strategies that involve vaccinating a target fraction (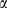) of susceptible individuals at each time step. After a round of vaccination, if the number of remaining susceptibles is less than a designated threshold (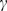), the vaccination campaign is discontinued. Policies defined in this way provide effective target population sizes, to which post-hoc corrections can be applied in light of knowledge of the population structure.

We assume that in a single time unit there is an upper bound on the maximum targetable fraction of susceptibles. In our simulations we set this bound to be 30%, so that several time units are required to vaccinate the majority of susceptibles. We also assume there is a period of time after the arrival of the initial infection before intervention can begin. In our examples, we assume this lag time to be 7 time units. These values are chosen purely for the purpose of demonstration, and can be assigned any value in the amei software.

**Figure 3:**
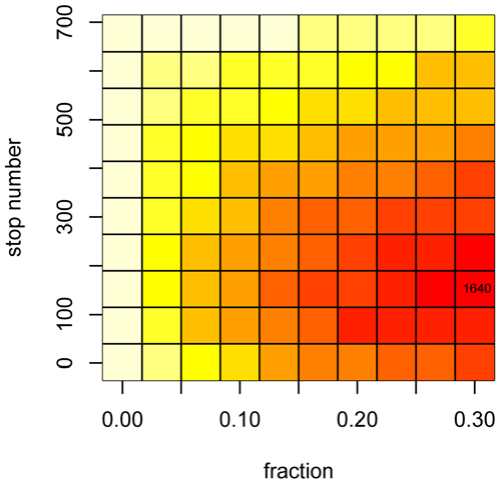
Expected cost surface for static interventions. The heatmap depicts the expected cost surface associated with variable stop time vaccination strategies based on the true parameter values. The minimum expected cost (1640 cost units) is achieved by a strategy of vaccinating 30% of susceptibles at each time step, until the number of susceptibles falls below 150. The maximum expected is realized through inaction (top row and left column policies are never implemented).

**Figure 4:**
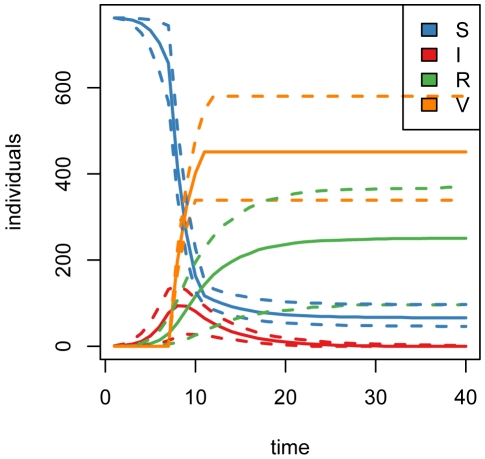
Simulated epidemics under static intervention. (2.5, 50, 97.5)-% quantiles for the numbers of susceptible, infected, recovered, and vaccinated individuals over 1000 simulations of the epidemic under the optimal variable stop time strategy based on true parameter values. The mean number of vaccine units dispensed is 442, with quantile bounds (339,580).

**Figure 5:**
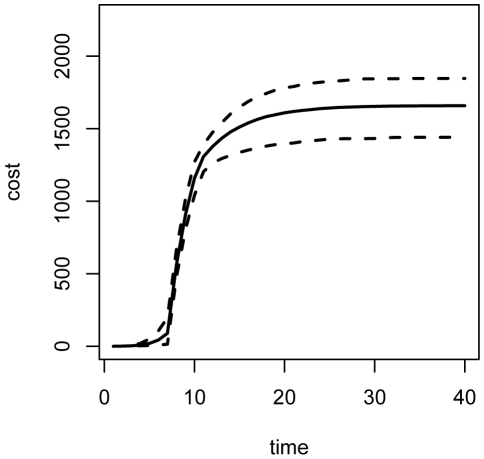
Expected costs under static intervention. Costs under optimal (2.5, 50, 97.5)-% quantiles for the total cost accrued over 1000 simulations of the epidemic under the optimal variable stop time strategy based on true parameter values. The mean total cost is 1652 cost units, with quantile bounds (1440,1846).

The optimal variable stop time vaccination strategy can be found by searching the policy space (i.e. pairs of fractions-to-vaccinate 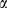 and stopping thresholds 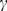) for the policy that most frequently produces the lowest expected cost. The calculation of the optimal policy therefore explicitly accounts for uncertainty associated with the disease transmission and recovery processes (see Methods) under a given valuation of the model parameters. Assuming a value of 2 cost units per dose of vaccine (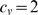), we use Monte Carlo simulation to estimate the expected cost surface associated with variable stop time policies based on the true parameter values (Figure 3). The minimum expected cost is achieved under a policy of maximum (30%) vaccination and a stopping threshold of 150 individuals. Repeated simulation of the epidemic under this policy shows that in the average case (dashed line), the policy amounts to 4 time units of maximum vaccination as soon as the initial lag is over (Figure 4). In situations where the number of susceptibles remaining after the lag is already below 150 individuals, no policy is implemented. The 95% central interval for the final distribution of total vaccine units dispensed is (339,581), representing variation in the total size of the epidemic at the time of the vaccination sweep, and the numbers of new infections after vaccination begins. Figure 5 shows the distribution of total costs accrued under this policy. After the end of the vaccination campaign, the uncertainty bands widen, representing variations in the costs associated with maintaining the remaining population of infected individuals until their natural recoveries. The mean total cost at time 40 is 1652 cost units, approximately a 21% reduction in total cost compared to no-intervention.

### Adaptive Management

The intervention calculated in the previous section represents a gold-standard for this particular scenario because the vaccination strategy was calculated using the same parameter values and the same SIR model formulation as the simulated disease process. In most settings it will be natural to regard the transmission model parameters as unknowns to be estimated from incoming count data describing the sizes of the susceptible, infected, and recovered subpopulations. In this section we describe the procedure for performing adaptive management of an emerging epidemic, in which we account for parameter uncertainty and its impact on vaccination strategies.

An epidemic can be effectively summarized by the disease state of the population (*i.e.* the current numbers of susceptible and infected individuals) and by the SIR model parameters that define the dynamics of transmission, death, and recovery. In adaptive management, the former is used to perform inference on the latter. Each time new data are collected, Markov chain Monte Carlo (MCMC) is used to sample from the current posterior distribution on model parameters. The optimal variable stop time strategy associated with each set of sampled parameter values is calculated, and the policy that most frequently minimizes the total expected cost (over all sampled parameter values) is enacted at the next time step. The fundamental difference between the adaptive policies calculated here and those calculated in the previous section is that here, the vaccination policy is a dynamic function of the current disease state and the current distribution of each parameter, whereas before, the policy was a static function of the initial disease state and the initial point estimate of each parameter.

The effectiveness of this approach can be similarly explored by repeated simulation of epidemics under adaptive management. As before, we assume an initial lag time of 7 time units before vaccination begins. Here we also introduce a cost associated with deaths (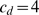). Even though the “true” model does not include mortality, the fitted model includes a mortality parameter (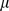). This allows examination of the degree to which adaptive management strategies are robust to model misspecification.

Initial uncertainty regarding parameter values is expressed in the form of vague/noninformative prior distributions, as specified in the Methods. The choice of prior distributions in Bayesian models is of fundamental importance, and other possible choices are mentioned in the Discussion section. At each time step, the state of the epidemic is advanced one time step using the same “true” parameter values used in the previous section. Intervention strategies, however, are calculated based on the current parameter estimates.

**Figure 6:**
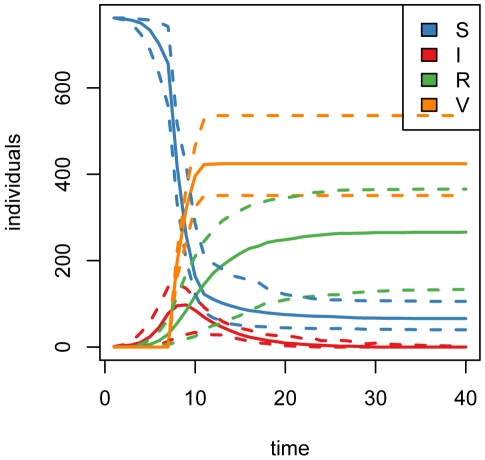
Simulated epidemics under adaptive management. (2.5, 59, 97.5)-% quantiles for the numbers of susceptible, infected, recovered, and vaccinated individuals over 100 simulations of the epidemic under optimal adaptive management. The mean number of vaccine units dispensed is 428, with quantile bounds (351,536).

**Figure 7:**
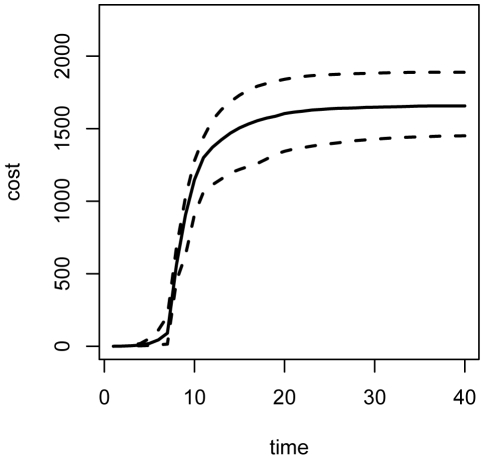
Expected costs under adaptive management. (2.5,50,97.5)-% quantiles for total cost accrued over 100 simulations of the epidemic under optimal adaptive management. The mean total cost is 1665 cost units, with quantile bounds (1450,1888).

**Figure 8:**
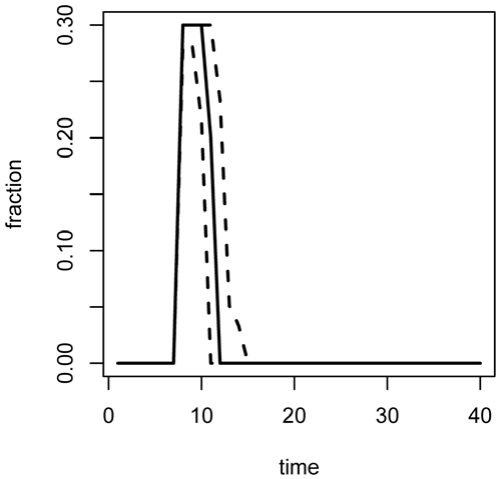
Vaccination levels under adaptive management. (2.5, 50, 97.5)-% quantiles for the fraction of susceptibles vaccinated at each time step over 100 simulations of the epidemic under optimal adaptive management.

**Figure 9:**
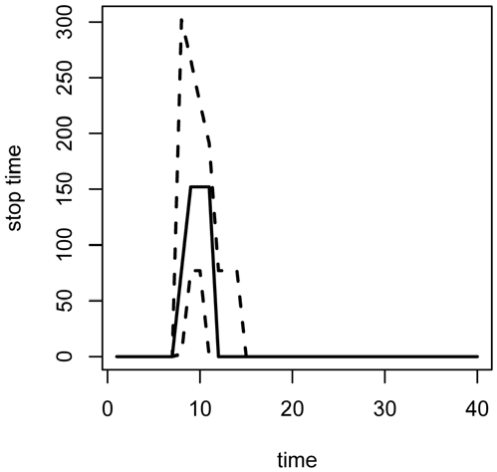
Stopping times under adaptive management. (2.5,50,97.5)-% quantiles for the policy stop time at each time step over 100 simulations of the epidemic under optimal adaptive management.

Figures 6 and 7 show the distributions of susceptible, infected, recovered, and vaccinated individuals, and total accumulated costs for repeated simulation of the epidemic under adaptive management. These dynamics can be compared to those in Figures 4 and 5 in order to explore the effect of propagation of parameter uncertainty on efficacy of control measures. Compared to Figure 4, the central 95% region associated with the total number of vaccine units dispensed over the course of the intervention is more compact: (351, 536) with mean of 428 units for the adaptive policy versus (339, 580) with a mean of 442 units for the nonadaptive policy. The tighter bound about a smaller mean is due to the ability of the adaptive strategies to methodically diminish the vaccination campaign as a function of the epidemic state. This can be seen in Figures 8 and 9, which display the distributions of implemented vaccination strategies for each time step during the course of adaptive management. In the average case (dashed line), the maximum policy is enacted for 3 time steps, followed by a round of 20% vaccination. The uncertainty surrounding the implemented strategies indicates the degree to which the the adaptive policies are adjusted in light of data. In epidemics associated with the upper 97.5 percentile of vaccination strategies (top solid line in Figures 8 and 9), the adaptive policy calls for 4 rounds of maximum vaccination followed by a round of 20% vaccination, followed by a final round of 5% vaccination. In this way, the adaptive nature of the interventions enables more efficient use of vaccine resources than achieved under nonadaptive policies.

**Figure 10:**
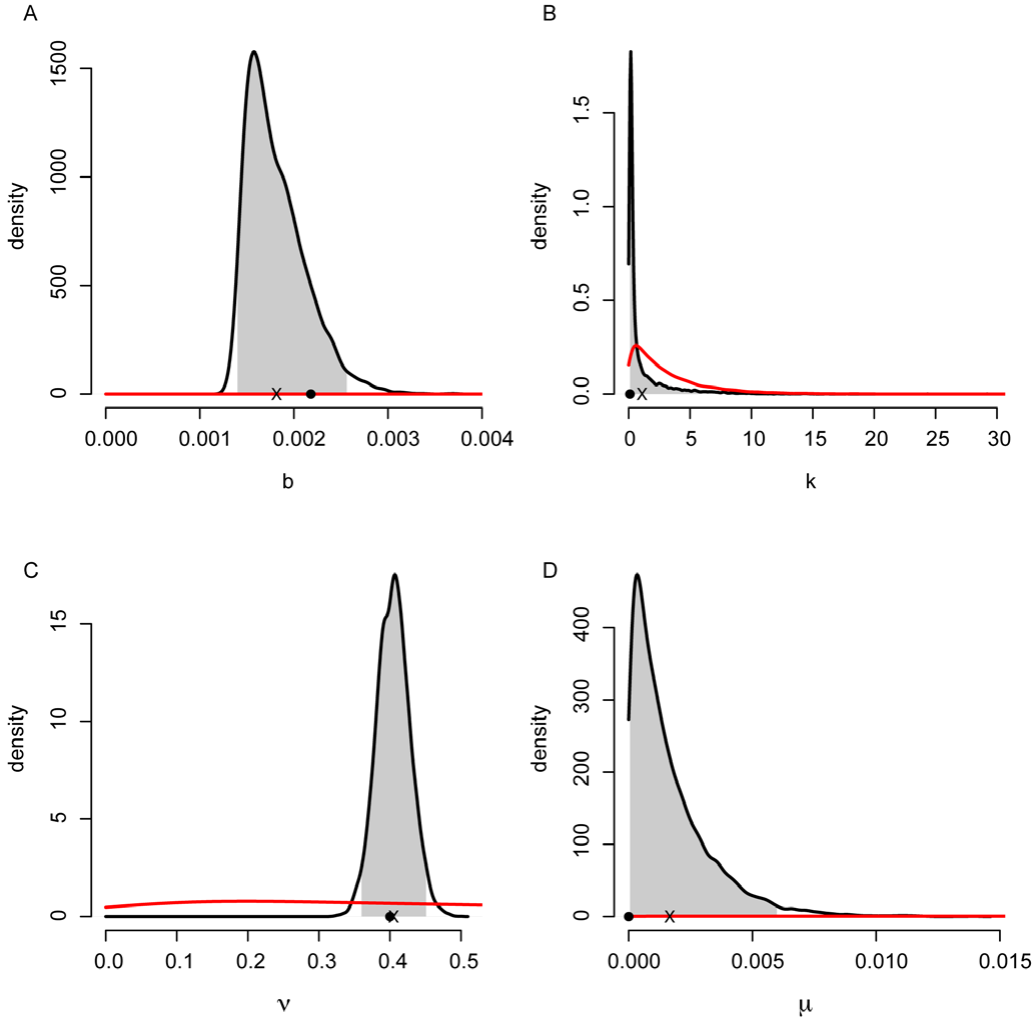
Online parameter estimates. Final posterior density estimates for the transmission rate (A), overdispersion parameter (B), recovery rate (C), and mortality rate (D). “True” parameter values are indicated by a dot, mean posterior values are indicated by an ‘x’, and the central 95% region of the distribution is shaded. Prior densities on the same regions are shown in red.

The distribution of total cost associated with the adaptive intervention simulations (Figure 7) is essentially equivalent to the distribution of costs achieved under static intervention with perfect information (Figure 5), indicating that even the short period of data collection prior to action produces parameter estimates that are sufficient for accurate prediction of the disease dynamics. Figure 10 shows the final posterior distributions on the four model parameters estimated from the data during one simulation of the epidemic under adaptive management. True values are indicated with a circle, mean values are indicated with an ‘x’, and the central 95% region of each distribution is shaded. The prior densities of each parameter for the same interval are shown in red. As mentioned above, the inference model is misspecified relative to the model being used to simulate the epidemic, in that the inference model includes a mortality parameter (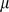, see **Methods**), even though no deaths were observed in the simulated outbreaks. By coupling the policy calculations with an inference framework, the effect of such model misspecification appears to be reduced.

We can further demonstrate the utility of the adaptive approach in situations of more severe model misspecfication. To do so, we construct a simulation experiment in which the inference model upon which the adaptive management is based is as described here, but in which the underlying transmission model through which new infecteds are generated is an entirely different, non-nested transmission model with a latent infective resevoir (see the amei vignette on CRAN [11] for details). This situation more closely resembles one that may be encountered in practice, where new infections are arising from an actual disease transmission process whose dynamics are at best approximated by any mathematical characterization. Table 1 compares summaries of the posterior distribution of cumulative cost arising under adaptive management to those predicted under the optimal static policy using parameters estimated for the misspecified model based on a completely observed epidemic. It is important to recognize that the adaptive policy is at a severe disadvantage, basing its actions on parameter estimates produced simultaneously during the course of a single epidemic (and using vague prior distributions) while the static policy conditions on parameter estimates obtained from a completely observed epidemic. In spite of this, the adaptive policy achieves nearly identical costs.

**Table 1:** Expected costs under model misspecification.

	2.5%-ile	Mean	Median	97.5%-ile
Adaptive	1910	2091	2089	2311
Nonadaptive	1888	2085	2085	2295

Comparison of adaptive and nonadaptive policy costs when the inference model is misspecified. Even though the static policy is based on parameter estimates obtained after a completely observed epidemic, the costs associated with adaptive management are similar.

We have now shown the near equivalence of the adaptive and static policies in two different scenarios. These situations indicate that the proposed methodology is efficiently and with sufficient accuracy estimating the parameters of the transmission model, such that adaptive strategies based on these on-line estimates produce equivalent outcomes to those static strategies based on full retrospective analyses. Moreover, it is simple to demonstrate that static control measures based on reasonable but imperfect parameter estimates can lead to substantially worse outcomes/higher costs than the adaptive policies (Table 2). In real situations, where actions must be based on parameter estimates made from incomplete or limited information, the practice of iterative refinement of estimates and policies is likely to result in significantly improved outcome.

**Table 2:** Expected costs under imperfect parameter estimates.

	2.5%-ile	Mean	Median	97.5%-ile
Adaptive	1451	1665	1657	1888
Nonadaptive	1938	2103	2100	2264

Comparison of adaptive and nonadaptive policy costs when static management is based on imperfect parameter estimates (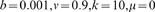).

## Discussion

We have demonstrated a novel adaptive management strategy based on a relatively simple characterization of the underlying SIR model and the epidemiological cost function. In principle, this methodological framework can readily accommodate more complicated disease dynamics such as immigration, latent infected states, missing data, and vector-communicated diseases, as well as more complicated intervention strategies that allow combined vaccination and quarantine. However, the incorporation of such features is likely to impose a heavy computational burden, and so model complexity should only be increased when additional parameters are supported (and identified) by the data and demanded by the biology. As in all Bayesian analyses, care must be taken when choosing prior distributions. In this study, our primary interests required the use of vague/noninformative prior distributions, in order to demonstrate the estimability of model parameters. In practice, informative, even pessimistic priors (i.e., overestimated infectiousness and mortality, underestimated recovery) may provide useful reference points for the adaptive policy calculations, especially in situations of acute infections for which the duration of the epidemic may be too short for incoming data to dominate the prior information. In such situations, the adaptive approach still provides the opportunity for data to inform parameter values if it becomes available, while basing interventions on current parameter estimates as determined by their prior distributions.

There is an important choice to be made in assigning costs to the various actions that comprise an intervention strategy. A monetary valuation scheme is the most straightforward, but it may be difficult to construct such a scheme that adequately represents all aspects of the decision. One alternative would be a valuation in which each cost is chosen to represent a probability of mortality. In this way, the cost to be minimized would be the expected total loss of life for the epidemic under a given intervention strategy. By assuming that the removal rate can be expressed as 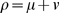, where 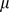 is the rate of disease-induced mortality and 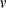 is the rate of natural recovery from the infected state, we can set 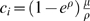, so that the cost associated with maintaining a given number of infected individuals for a unit of time is the number of infected individuals that are expected to die in a unit of time. Similarly, situations exist where it is reasonable to assign a probability of mortality to the removal of susceptibles, as in the cases of smallpox vaccination or the culling of livestock.

A related extension to this framework would involve applying a monetary constraint to a loss-of-life cost function. If we were to assume 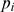 and 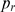 to be, respectively, the probabilities of mortality associated with untreated infected individuals and the removal of susceptibles, and define *d* to be the monetary resources available for the intervention, then within this framework it is possible to find the intervention that minimizes the total loss-of-life subject to the total spending constraint *d*. Similarly, it would be possible to optimize with respect to some selective criterion in order to preserve vaccine efficacy rather than select unnecessarily for drug-resistant pathogens. Also note the possibility of calculating policies based on minimization of some quantile of the realized cost rather than the mean cost. This would lead to minimization of costs associated with worst case scenarios, rather than that associated with the average case scenario. These and other alternative formulations of the underlying optimization problem can be easily accommodated in the framework presented here.

The utility of adaptive interventions is especially evident in situations of an emerging pathogen with which the host population has no previous experience. In such a situation, vaccines will not be immediately available at the onset of the epidemic, and so a methodology for combining currently available actions while anticipating the future availability of vaccines would be of great use. Effective epidemiological intervention requires swift decision in consideration of the various direct and indirect costs of intervention. The methodological framework described here provides a decision theoretic basis for automating this process.

## Materials and Methods

All statistical and computational methodology described here has been implemented in a freely available R package called amei (Adaptive Management of Epidemiological Interventions), which can be downloaded at http://cran.r-project.org/web/packages/amei/index.html [11].

### SIR Model

We consider a standard Susceptible-Infected-Removed (SIR) model [10], [12] with no loss of immunity but with mortality. In this model, the dynamic variables at time *t* are the number of susceptible individuals, *S*(*t*); the number of infected individuals, *I*(*t*); the number of recovered individuals, *R*(*t*); and the number of removed/dead individuals, *D*(*t*). We assume that the population is closed to immigration such that *S*(*t*)+*I*(*t*)+*R*(*t*)+*D*(*t*) = *N* is constant, and any three of the dynamic variables define the fourth.

To characterize the transmission of the disease, we adopt the negative binomial form for the transmission function [13], so that the model parameters are the transmission rate *b*, the overdispersion parameter *k*, the death rate μ, and the rate of recovery to the immune class ν. Under these assumptions, the SIR model is described by the following system of differential equations [12], [13]: 
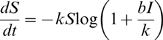
(1)

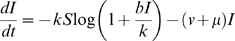
(2)

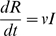
(3)

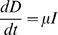
(4)The negative binomial transmission function implies that disease transmission occurs following a Poisson process in which encounters between infected and susceptible individuals are Poission distributed with the encounter rate varying according to a gamma distribution with coefficient of variation 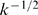. Via the parameter *k*, the negative binomial transmission function can account for social interactions and/or network factors in disease transmission, without requiring explicit characterization of the population structure.

The SIR model formulation also leads immediately to a natural discrete time approximation for the numbers of infections (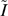), recoveries (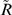) and deaths (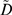) arising in the unit time interval from *t* to *t*+1. Holding the total number of infected individuals *I* constant and integrating Equation 1 over a unit time interval gives 
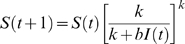
(5)so that the fraction of susceptible individuals surviving a unit time interval is 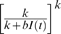 Viewed as a discrete time stochastic process, the number of new infections occurring between time *t* and *t*+1 when *S*(*t*) = *s* and *I*(*t*) = *i* can be described by
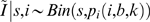
(6) where 
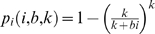
and *Bin*(*n*,π) is the standard binomial distribution. Similarly, by integrating Equations 3 and 4, we have that the numbers of recoveries and deaths occurring between time *t* and *t*+1 can be described by
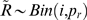
(7)

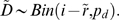
(8)where 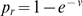 and 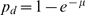 The forward dynamics for the total numbers of susceptible and infected individuals are therefore individuals are therefore
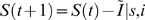
(9)

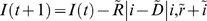
(10)Here lower case denotes the realized value of the associated capital letter random variable. In this discrete time approximation we have assumed a particular ordering of events, namely that recoveries occur first, followed by deaths from among those infected individuals who did not recover, followed by new infections. Simulation studies indicated that these assumptions, as well as other possible orderings, resulted in system dynamics that were approximately equal in expectation to deterministic solutions of the continuous time SIR model.

In all forward simulations of the disease dynamic (except where noted) we assume the “true” underlying parameter values to be those estimated by Murray [9], with the exception of the negative binomial overdispersion parameter *k*. Thus, *b* = 0.00218, ν = 0.4, and μ = 0 (no disease-related mortality). We set the overdispersion parameter to be *k* = 0.1, in order to produce epidemics that, without intervention, have run their course by 40 time units but such that there is variation in the size of the outbreak.

### Epidemiological Cost Function

We formulate the total expected cost of the epidemic in terms of the underlying costs associated with maintaining infected individuals until recovery, suffering death, and administering vaccinations. Let 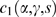 denote the cost associated with interventions involving susceptibles when *S*(*t*) = *s*. Here α is the fraction of susceptibles that are moved directly into an immune/recovered class, as by vaccination, and γ is the threshold below which the intervention is discontinued. Letting 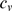 denote the cost per unit, then
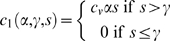
(11)We let 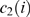 denote the cost associated with interventions involving infecteds when *I*(*t*) = *i*. This component includes the costs associated with maintaining the non-recovered infected individuals and costs associated deaths, as in 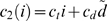
(12)where 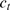 is the cost per treatment/maintenance of a non-removed infected individual, and 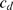 is the cost per death.

Assuming the initial epidemiological state is 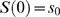 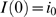 the expected total cost of the epidemic under intervention strategy (α,γ) can be expressed recursively as
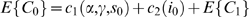
(13)where 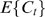 denotes the expected cost accumulated from time *t* onwards. The optimal intervention strategy (α,γ) is the one that minimizes the total accumulated cost over the course of the epidemic. Two methods for calculating such strategies are as follows.

### Calculating Variable Stop Time Vaccination Strategies

The total expected cost depends on the parameter values and the initial epidemiological state 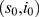. Thus, conditional on a set of parameter values, Monte Carlo simulation can be used to search over values of α and γ in order to find the combination that minimizes 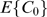 For each combination of α and γ, with α ranging from 0 to 0.7 and γ from 0 to 750 in increments of 50, we conduct 100 simulations of the epidemic, using the true parameter values, in order to estimate the mean cost associated with the intervention (α,γ). The strategy producing the lowest mean cost is defined to be the optimal intervention.

### Calculating Adaptive Management Strategies

As above, the expected cost surface associated with a given set of parameter values (as obtained by MCMC, described below), can be explored using standard Monte Carlo methods. At each time step, MCMC is used to produce 10,000 samples from the current posterior distribution on model parameters. These samples are thinned to 100 samples, and for each of these 100 samples the optimal variable stop time vaccination strategy is calculated as described above. The adaptive strategy to be implemented at that time step is defined to be the most frequently optimal strategy for the 100 posterior samples.

Notice that if we were to allow the fraction of the population targeted for vaccination to be a function of future disease states, rather than a static fraction and a stopping threshold, we could regard Equation 13 as a stochastic iteration equation and use stochastic dynamic programming [14] to calculate the optimal intervention associated with a set of parameter values. Such an approach may be useful for situations in which knowledge of the disease state is available, but for whatever reason sequential inference is not possible. In the situation considered here, in which the static strategy is sequentially updated based on the current disease state and parameter estimates, the adaptive strategy that emerges is similarly flexible, in that it consists of a state-dependent sequence of target fractions, but does not involve the additional computational burden associated with stochastic dynamic programming.

### Online Parameter Estimation

We use Markov Chain Monte Carlo (MCMC) [15] to learn about the posterior distribution of of *b*, *k*, ν, and μ conditioned on the evolution of the epidemic observed so far. The likelihood is given recursively in Equations 6–8. Assume, at first, that no intervention strategy is implemented. Let 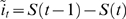 be the number of new infecteds at time *T*, and similarly for the newly recovered and dead individuals 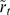 and 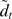 so that 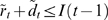 Then, the likelihood up to *T* is given by
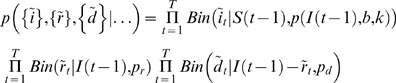
(14)and we can see that it consists of three mutually independent components.

Conditional conjugacy can be exploited for ν and μ via Beta priors for 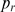 and 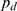. A Beta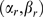 prior for 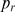 implies that 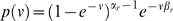
(15)Conjugate updating leads to the posterior conditional 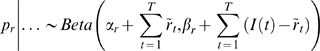
(16)The form of the conditional posterior for ν is similar to Equation 16 and can be simulated by first drawing 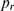 via Equation 16 and then applying the inverse transformation 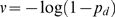. Sampling for μ proceeds similarly with 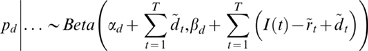
(17)So it is possible to take Gibbs samples for ν and μ so long as appropriate 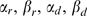 can be found to represent our prior beliefs. In ignorance we simply set these to unity, leading to uniform priors on 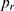 and 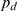.

Obtaining samples for *b* and *k* requires the Metropolis–Hastings algorithm. Our prior beliefs can be encoded with gamma distributions, and conditional on a previous sample (*b*,*k*) the next sample (*b*′,*k*′) can be obtained by Metropolis-within-Gibbs steps using: 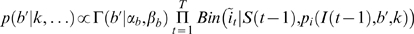
(18)

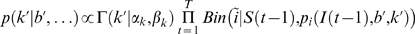
(19)For the prior settings, we currently use 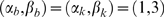 which (though seemingly informative at first glance) turns out be uninformative on the scale of the support of the posterior. We find that random walk uniform proposals on the positive real line, i.e., *b*′∼*U*[3*b*/4,4*b*/3], gives reasonably good mixing from the Markov chain, as evidenced by visual inspection of parameter traces and other convergence diagnostics. More details pertaining to technical issues such as MCMC convergence appear in the amei vignette [11].

The presence of a vaccination strategy necessitates a simple change to the above equations. Replace *S*(*t*−1) with 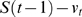, where 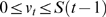 is the number of susceptibles which have been vaccinated. Then 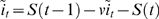.
